# BET Protein Inhibitor JQ1 Modulates Mitochondrial Dysfunction and Oxidative Stress Induced by Chronic Kidney Disease

**DOI:** 10.3390/antiox12051130

**Published:** 2023-05-20

**Authors:** Sandra Rayego-Mateos, Pamela Basantes, José Luis Morgado-Pascual, Beatriz Brazal Prieto, Beatriz Suarez-Alvarez, Alberto Ortiz, Carlos Lopez-Larrea, Marta Ruiz-Ortega

**Affiliations:** 1 Cellular Biology in Renal Diseases Laboratory, IIS-Fundación Jiménez Díaz-Universidad Autónoma Madrid, 28040 Madrid, Spain; pamelabasantes@hotmail.com (P.B.); biomorgui@hotmail.com (J.L.M.-P.); beatrbraz@gmail.com (B.B.P.);; 2 Ricors2040, 28029 Madrid, Spain; bea230@hotmail.com (B.S.-A.); aortiz@fjd.es (A.O.);; 3 Maimonides Biomedical Research Institute of Cordoba (IMIBIC), University of Cordoba, 14004 Cordoba, Spain; 4 Translational Immunology, Instituto de Investigación Sanitaria del Principado de Asturias (ISPA), Hospital Universitario Central de Asturias, 33011 Oviedo, Spain; 5 Division of Nephrology and Hypertension, IIS-Fundación Jiménez Díaz-Universidad Autónoma Madrid, 28040 Madrid, Spain

**Keywords:** mitochondrial dysfunction, BET proteins, JQ1, renal damage, mitochondrial dynamics, mitophagy

## Abstract

Among the mechanisms involved in the progression of kidney disease, mitochondrial dysfunction has special relevance. Epigenetic drugs such as inhibitors of extra-terminal domain proteins (iBET) have shown beneficial effects in experimental kidney disease, mainly by inhibiting proliferative and inflammatory responses. The impact of iBET on mitochondrial damage was explored in in vitro studies in renal cells stimulated with TGF-β1 and in vivo in murine unilateral ureteral obstruction (UUO) model of progressive kidney damage. In vitro, JQ1 pretreatment prevented the TGF-β1-induced downregulation of components of the oxidative phosphorylation chain (OXPHOS), such as cytochrome C and CV-ATP5a in human proximal tubular cells. In addition, JQ1 also prevented the altered mitochondrial dynamics by avoiding the increase in the DRP-1 fission factor. In UUO model, renal gene expression levels of cytochrome C and CV-ATP5a as well as protein levels of cytochrome C were reduced These changes were prevented by JQ1 administration. In addition, JQ1 decreased protein levels of the DRP1 fission protein and increased the OPA-1 fusion protein, restoring mitochondrial dynamics. Mitochondria also participate in the maintenance of redox balance. JQ1 restored the gene expression of antioxidant proteins, such as Catalase and Heme oxygenase 1 in TGF-β1-stimulated human proximal tubular cells and in murine obstructed kidneys. Indeed, in tubular cells, JQ1 decreased ROS production induced by stimulation with TGF-β1, as evaluated by MitoSOXTM. iBETs, such as JQ1, improve mitochondrial dynamics, functionality, and oxidative stress in kidney disease.

## 1. Introduction

Chronic kidney disease (CKD) is a common progressive condition estimated to become the fifth global of cause death by 2040, mainly due to population aging and the increasing prevalence of risk factors such as obesity, diabetes and hypertension [1].

Mitochondria are complex intracellular double-membrane organelles that supply energy to kidney tubular cells to support their transport functions [2,3]. The mitochondrial membrane potential (∆Ψm) can be altered during kidney damage [4,5,6]. The ∆Ψm is generated by proton release by complexes I, III and IV of the electron transport chain (ETC) during oxidative phosphorylation (OXPHOS) that also produces adenosine triphosphate (ATP) [7]. Other studies described that the mitochondria also participates in redox and iron homeostasis and regulates inflammation, cell death, and danger signaling [8].

Mitochondrial damage has been related to the progression of several diseases, including CKD [9,10,11,12]. Mitochondria have quality control systems such as the unfolded protein response, antioxidant defense and the heat shock response (HSR) that, together with a balanced turnover, contribute to maintain healthy mitochondria [13]. Mitochondrial turnover depends on mitochondrial biogenesis, mitophagy (a specific form of autophagy that removes damaged mitochondria) and mitochondria dynamics. Mitochondria dynamics represents the equilibrium between mitochondria fission (division of mitochondria into two daughter organelles) and fusion (two mitochondria merge their membranes to create a larger organelle) to create a functional and complete organelle [9,14,15,16,17]. Mitochondria can regulate their morphology, creating a tubular network coordinated by fission and fusion events to adapt their size, shape, and distribution to modify extracellular and intracellular environment. The balance between fission and fusion processes has been described as “mitochondrial dynamics” [18]. Mitochondrial fusion induces the combination of individual mitochondria through their membranes, the outer (OMM) and the inner mitochondria membranes (IMM). Outside, the mitochondrial membrane components such as Mitofusin 1 and 2 (MFN1 and MFN2) regulate outer membrane fusion, and protein optic atrophy 1 (OPA1) regulates inner membrane fusion and cristae remodeling [19,20,21,22]. On the other hand, mitochondrial fission takes place when this organelle is divided into two separate mitochondria, a process modulated by Dynamin-related protein 1 (DRP1). DRP1 is located in the cytosol, and during fission, it is recruited to the mitochondria to regulate membrane constriction in a GTP-dependent manner [23]. In renal disease, unbalanced fission and fusion may trigger organelle fragmentation through excess fission or hypertubulation through excess fusion [14].

Mitophagy also collaborates in mitochondrial turnover. Mitophagy is mainly mediated by the PTEN Induced Kinase 1 (PINK1)/Parkin RBR E3 Ubiquitin Protein Ligase (PARK2) pathway that, by removing damaged mitochondria, protects tissues from inflammation and disease [14,16].

DNA- or histone-related epigenetic modifications contribute to renal damage [24]. Epigenetic drugs have been proposed as therapeutic options in kidney diseases [25,26]. Among them, the inhibitors of bromodomain (BRD) and extra-terminal (BET) proteins (iBETs) have been shown to exert beneficial effects in experimental pathologies [27,28,29], including kidney diseases. The BET family of proteins are “readers” of epigenetic marks and contains a pair of two N-terminal BRD known as BD1 and BD2 [26]. The BET proteins interact with transcription factors via BRD in nucleus in order to promote their close-up with enhancer or super-enhancer in the active zones of chromatin and regulate their function; this mechanism represents an indirect way of regulating gene transcription. However, BET proteins can also directly regulate transcription by interaction with “remodelling proteins” to create changes in chromatin structure. This mechanism is described by BRD4, the most studied member of the family. BRD4 recruits proteins via its extra-terminal domain (ET), generating a complex with other proteins, phosphorylating polymerase II and initiating the transcription [26].

The BET inhibitor (iBET) JQ1 has been shown to ameliorate inflammation and fibrosis in different mice models of renal damage by the direct inhibition of proinflammatory genes such as CCL2 and IL-17 or blocking the activation of transcription factors such as NF-KB, SOX-9 or RORγT [28,30,31]. Recent data suggest that iBETs exert beneficial effects in cardiovascular damage and tumors, potentially through modulation of mitochondrial dysfunction [32,33,34]. However, there are no studies in kidney diseases on the impact of iBETs on mitochondria.

This study aimed to evaluate whether iBETs could modulate mitochondrial dysfunction in tubular epithelial cells and in experimental renal injury. To this aim, the different processes involved in mitochondrial damage including the regulation of mitochondrial dynamics and mitophagy, and the role of redox processes were evaluated.

## 2. Materials and Methods

### 2.1. Ethics Statement

All animal procedures were performed in 3-month-old male C57BL/6 mice according to the guidelines of animal research in the European Community and with prior approval by the Animal Ethics Committee of the Health Research Institute IIS-Fundación Jiménez Díaz.

### 2.2. Unilateral Ureteral Obstruction (UUO) Model

Studies were performed in adult male C57BL/6 mice (9–12 weeks old, 20 g; obtained from Harlan Interfauna Ibérica, S.A., Barcelona, Spain) and maintained at the local animal facilities, with free access to food and water, normal light/dark cycles, and under special pathogen-free conditions. The BET bromodomain inhibitor (iBET) JQ1, a thieno-triazolo-1,4-diazepine, was synthesized and provided collaboratively by Dr. James Bradner (Dana-Farber Cancer Institute, Boston, MA) [35]. For in vivo studies, JQ1 was dissolved in 10% hydroxypropyl β-cyclodextrin in sterile H_2_O and used at a therapeutic dose (100 mg/kg/day, i.p.) as previously described [28].

Unilateral ureteral obstruction (UUO) was performed under isoflurane-induced anesthesia using vaporizer equipment at an optimal dose of 4.5% for induction; 1–2% for maintenance/inhalation route. The left ureter was ligated with surgical thread (5/0) in two positions, and a cut was made between them to prevent urinary tract infection according to the protocol [36]. The contralateral left kidney was used as control (C) for the right obstructed kidney (Ob). The animals were administered with analgesics for 3 days after surgery (buprenorphine, 0.1 mg/kg/day by subcutaneous injection) to improve post-operative recovery

Mice (*n* = 6–7 per group) were treated daily with JQ1 100 mg/kg/day, from 1 day before the intervention until the moment of sacrifice, 2 and 5 days later. Mice were anesthetized with 5 mg/kg xylacin (Rompun, Bayer AG, Leverkusen, Germany) and 35 mg/kg ketamine (Ketolar, Pfizer, New York, NY, USA). After this point, kidney samples were perfused and subsequently extracted to be frozen in liquid nitrogen for subsequent RNA and protein extraction.

### 2.3. Cell Culture

HK2 cells (human kidney proximal tubule epithelial cells) were grown in the RPMI medium (Gibco Roswell Park Memorial Institute) with 10% FBS, 100 U/mL penicillin, 2 mM glutamine (Sigma-Aldrich, St. Louis, MO, USA), 5 mg/mL insulin–transferrin–selenite (ITS), hydrocortisone (36 ng/mL) (Sigma-Aldrich, St. Louis, MO, USA) and 100 μg/mL streptomycin at 37 °C in 5% CO_2_. Subconfluent cells (60,000 cells/cm^2^) were incubated with stimuli in a serum-free medium for 24 h. Some cells were pretreated with JQ1 solved in DMSO (0.25%) (provided by Dr Brandner of the Danna Farber cancer institute, Boston, MA, USA) at 5 μM for one hour prior to stimulation with TGF-β1 (Preprotech, Cranbury, NJ, USA). Viability studies with different doses of JQ1 had previously established an effective dose with minimal toxicity. In addition, 0.25% DMSO was included as the JQ1 solvent control, and non-toxicity in this condition was observed. TGF-β1 (10 ng/mL) was maintained for 24 or 48 h at 37 °C until the end of the experiment.

### 2.4. Study of Proteins

Total protein from frozen renal tissue was isolated and homogenized in a lysis buffer (50 mmol/L Tris-HCl, 150 mol/L NaCl, 2 mmol/L EDTA, 2 mmol/L EGTA, 0.2% Triton X-100, 0.3% IGEPAL, 10 μL/mL proteinase inhibitor cocktail, 0.2 mmol/L PMSF, and 0.2 mmol/L orthovanadate) with the protocol described in [28]. In the case of nuclear fractions, they were separated from renal tissues using the NE-PER Reagent kit following the manufacturer’s instructions (Thermo Fisher, Waltham, MA, USA). Renal tissues were homogenized and incubated with two reagents (CER I and CER II with protease inhibitors to maintain extract integrity and function) that generate tissue pellet that causes cell membrane disruption and the release of cytoplasmic contents. After recovering the intact nuclei from the cytoplasmic extract by centrifugation, the proteins were extracted out of the nuclei with the third reagent (NER I). Proteins (20–100 μg per lane, quantified using a BCA protein assay kit) were separated on 8–12% polyacrylamide-SDS gels under reducing conditions [28]. Samples were then transferred onto polyvinylidene difluoride membranes (Thermo Scientific, Waltham, MA, USA), blocked with TBS/5% non-fat milk/0.05% Tween-20, and incubated overnight at 4 °C with the antibodies against the following proteins (dilution): ATP5A (1:1000, sc-136178 Santa Cruz, Santa Cruz, CA, USA), DRP-1 (1:1000, sc-271583 Santa Cruz, Santa Cruz, CA, USA); Cytochrome C (1:1000, sc-13156, Santa Cruz, Santa Cruz, CA, USA); PINK 1 (1:1000, sc-517353 Santa Cruz); OPA-1 (1:1000, sc-393296 Santa Cruz, Santa Cruz, CA, USA); LC3B (1:1000, NB100-2220, Nobus Biological, Centennial, CO, USA); TFEB (1:1000, sc-166736 Santa Cruz, Santa Cruz, CA, USA). After, the membranes were incubated with peroxidase-conjugated IgG secondary antibody. Subsequently, the membranes were developed using an ECL chemiluminescence kit (Amersham; Buckinghamshire, UK). Loading controls were performed using an anti-GAPDH antibody (1:5000; CB1001, Millipore, Burlington, MA, USA); ERK2 (1:1000, Santa Cruz, Santa Cruz, CA, USA); α-tubulin (1:5000, T5168, Sigma, Burlington, MA, USA) and Histone H3 (1:1000, sc-56616 Santa Cruz, Santa Cruz, CA, USA) for nuclear extracts. The results were analyzed by LAS 4000 and Amersham Imager 600 (GEHealthcare, Chicago, IL, USA) and densitometered by the Quantity One software (Biorad, Hercules, CA, USA).

### 2.5. Gene Expression

Total RNA samples from kidney cells or tissue were isolated with TRItidy GTM (PanReac; Darmstadt, Germany). Next, 2 µg of total RNA was used to synthesize cDNA by a High Capacity cDNA kit (Applied Biosystems, Foster City, CA, USA). Then, quantitative gene expression analysis was conducted by real-time PCR (fast real-time AB7500 system; Applied Biosystems, Foster City, CA, USA) using the MGB TaqMan fluorogenic probes and pre-designed primer TaqMan Gene Expression Assays (accession number; reference): *Dnm1* (NM_001276341.1; Mm01342914_m1); *Map1lc3b/Lc3b* (NM_025735.3; Mm00458724_m1) *Opa1* (NM_007505.2; Mm00431960_m1); *Pink1* (NM_026880.2; Mm00550827_m1) and *Gapdh* (NM_001289726.1; Mm99999915_g1) and other probes from IDT: Cycs (NM_007808.4; 229849510), CAT (NM_009804.2; 22984990), HMOX-1 (NM_010442.2;229849478). The mRNA copy number was calculated for each sample with the software present on the instrument using the Ct value. The results were expressed as a copy number and were obtained relative to unstimulated control samples. For the quantification, the qualitative method of comparison with the expression of constitutive GAPDH was used.

### 2.6. Mitochondrial Viability

Mitochondrial viability in HK2 cells was assessed by MitoTracker^TM^. This kit contains a MitoTracker^TM^ Red CMXRos probe that crosses the cell membrane and binds to active mitochondria detecting changes in mitochondrial membrane potential. Cells were plated onto glass coverslips into the p24 plate and incubated for 15–45 min with the probe and subsequently incubated again with the wash buffer. Nuclei were counterstained with DAPI Images. Then, the samples were observed under a fluorescence confocal microscope (SP5, Leica Microsystems, Spain) at 585 nm. Images from four randomly chosen fields per glass coverslip (20–40X objective) were obtained in a blind manner independent of cells conditions. Negative controls included non-specific immunoglobulin and no primary antibody.

### 2.7. Superoxide Anion Production from Mitochondria

The MitoSOX^TM^ probe (Thermo Fisher, Waltham, MA, USA) was used to determine mitochondrial superoxide formation. Cells were incubated with the 2 mL 5 μM MitoSOX^TM^ reagent for 10 min at 37 °C in the dark. After, cell fluorescence was measured in the TECAN infinity 200 PRO multimode plate reader [using 488 nm (excitation) and 570 nm (emission) lasers].

### 2.8. Mitochondrial Membrane Potential

To determine the mitochondrial membrane potential, fluorescent labeling with TMRM (tetra-methyl-rhodamine) from Invitrogen (Thermo Fisher, Waltham, MA, USA) was used. Cells were incubated with the 2 mL 15 nM TMRM ™ reagent for 30 min at 37 °C in the dark. Then, the cells were washed and prepared for measurement on the TECAN infinity 200 PRO multimode plate reader [using 488 nm (excitation) and 570 nm (emission) lasers].

### 2.9. Statistical Analysis

Results are expressed as mean ± SEM of the n-fold increase with respect to the control (represented as 1). In the UUO model, data were obtained normalizing the UUO and UUO + JQ1 kidneys versus the contralateral kidney average. The Shapiro–Wilk test was used to evaluate sample normality distribution. For samples following the Gaussian distribution, a one-way ANOVA, followed by the corresponding post hoc analyses of Fisher’s LSD test, was used. To compare non-parametric samples, a Kruskal–Wallis and subsequent post hoc analysis of Uncorrected Dunn’s test was performed. Statistical analysis was conducted using GraphPad Prism 8.0 (GraphPad Software, San Diego, CA, USA). Values of *p* < 0.05 were considered statistically significant.

## 3. Results

### 3.1. JQ1 Prevented the Loss of Mitochondrial Viability Induced by TGF-β1 in Human Tubular Cells

**Figure 1:**
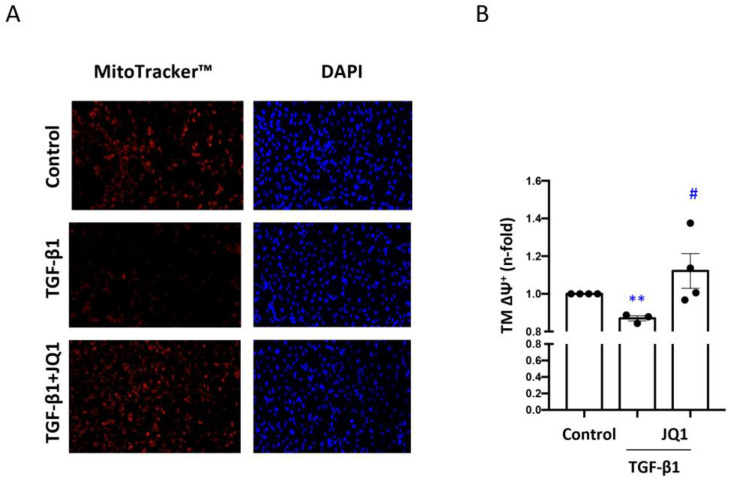
JQ1 modulates mitochondrial viability (**A**) and mitochondrial membrane potential (**B**) in human tubular epithelial cells. (**A**) HK2 cells were stimulated with TGF-β1 for 24 h to induce mitochondrial damage. Some cells were pretreated with JQ1 (5 μM). (**A**) Fluorescence microscopy images obtained following MitoTracker™ Red FM staining. Viable mitochondria appear red. (**B**) Measurement of mitochondrial membrane potential by fluorescence staining with TMRM (tetra-methyl-rhodamine) Invitrogen™ staining. Data are expressed as the mean ± SEM of 5 independent experiments. ** *p* < 0.01 vs. control; # *p* < 0.05 vs. TGF-β1.

First, we analyzed in vitro the effect of BET inhibition on mitochondrial viability in tubular epithelial cells. To induce mitochondrial damage, human tubular cells (HK2 cells) were stimulated with TGF-β1 for 24 h. To inhibit BET proteins, the cells were pretreated with the iBET JQ1 at a dose of 5 μM. Active mitochondria were identified by MitoTracker™ labeling that detects the membrane potential difference of intact mitochondrial membranes. A high number of active mitochondria were observed in untreated tubular cells (control) (Figure 1A), whereas in the TGF-β1-stimulated cells, there was a marked decrease in labeled mitochondria. Importantly, JQ1 pretreatment prevented the decrease in healthy mitochondria caused by TGF-β1 (Figure 1A).

### 3.2. JQ1 Prevented the Loss of Mitochondrial Membrane Potential (∆Ψm) and Expression of Components of the Electron Transport Chain (ETC) Induced by TGF-β1 in Human Tubular Cells

Mitochondria are characterized by the ∆Ψm which can be altered in injured tubular cells [37]. The ∆Ψm was determined by assessing the energy state of mitochondria using fluorescent labeling with tetramethylrhodamine (TMRM). The results showed significantly higher levels of the mitochondrial membrane potential in HK2 cells stimulated with TGF-β1 for 24 h and pretreated with JQ1 compared to those of cells exclusively stimulated with TGF-β1 (Figure 1B).

**Figure 2:**
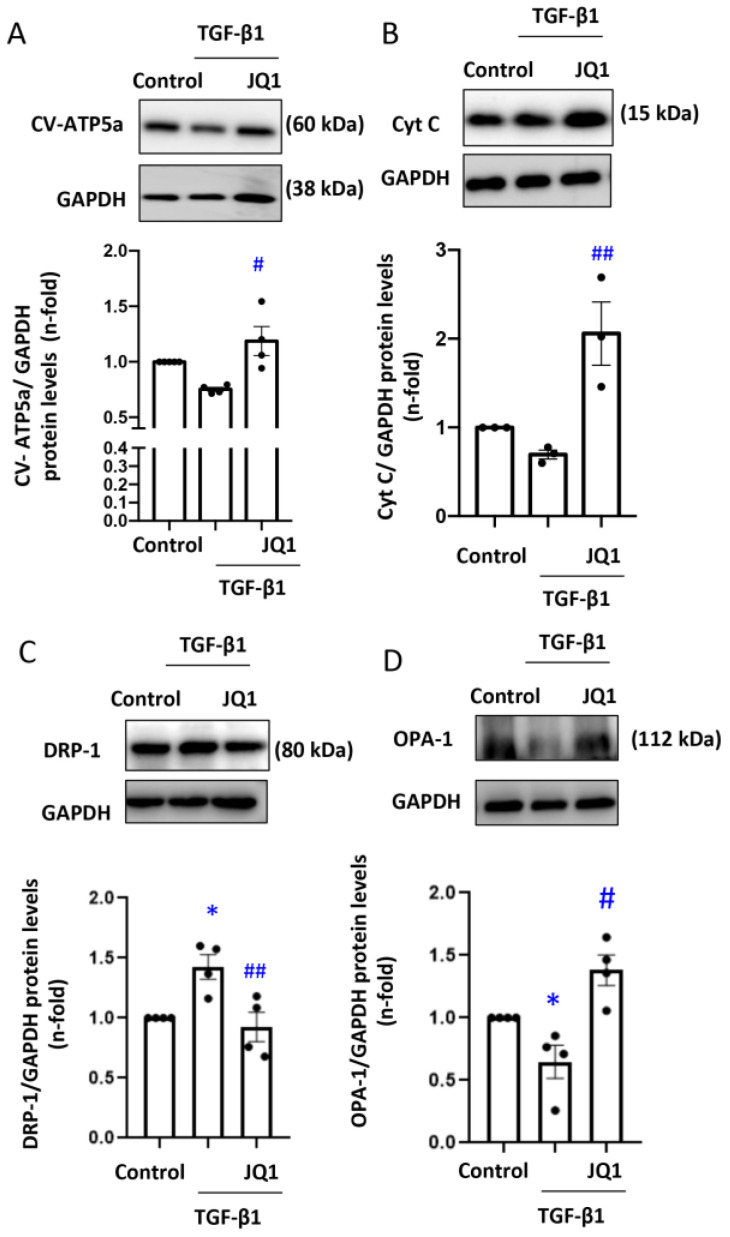
JQ1 modulates the mitochondrial ETC function and mitochondrial dynamics in human tubular epithelial cells. HK2 cells were stimulated with TGF-β1 cells for 24 h to induce mitochondrial damage. Some cells were pretreated with JQ1 (5 μM). (**A**) CV-ATP5, (**B**) Cyto C, (**C**) DRP-1 and (**D**) OPA-1 protein levels assessed by Western blot analysis. * *p* < 0.05 vs. control; # *p* < 0.05; ## *p* < 0.01 vs. TGF-β1.

The ∆Ψm potential is generated by protons released by complexes I, III and IV of the ETC during OXPHOS [38]. To evaluate changes in ETC components, the protein levels of the α subunit of the mitochondrial ATPase complex V (CV-ATP-5a) and cytochrome C (CytC) were determined by Western blot analysis. After exposure to TGF-β1, CV-ATP-5a and CytC protein levels decreased, and this was prevented by pre-treatment with JQ1 (Figure 2A,B).

### 3.3. JQ1 Prevented Dysregulated Mitochondrial Dynamics Induced by TGF-β1 in Human Tubular Cells

The expression of proteins involved in mitochondrial fusion–fission is altered during damage generating an imbalance in mitochondrial dynamics [17]. To determine the role of BET protein modulation in the fusion–fission imbalance in kidney cells, protein levels of DRP-1 and OPA-1 were measured in tubular cells stimulated with TGF-β1 for 24 h. TGF-β1 increased DRP1 and diminished OPA-1 protein levels, and this was prevented by pretreatment with JQ1 (Figure 2C,D).

### 3.4. JQ1 Decreased Oxidative Stress, Including Mitochondrial Oxidative Stress, Induced by TGF-β1 in Human Tubular Cells

**Figure 3:**
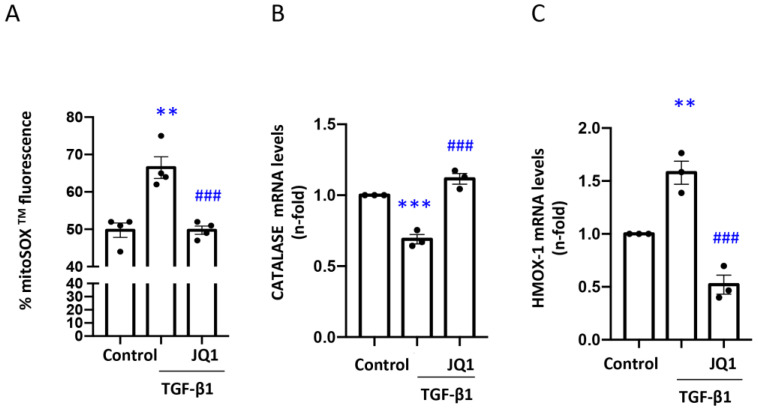
JQ1 decreases oxidative stress of mitochondrial origin in tubular cells stimulated with TGF-β1. (**A**) Measurement of fluorescence levels obtained by MitoSox™ staining in cells stimulated with TGF-β1 for 24 h and treated with JQ1 (5 μM). (**B**,**C**) Expression of (**B**) Catalase (CAT) and (**C**) heme oxygenase-1 (HMOX-1) mRNA levels determined by RT-PCR. Data are expressed as the mean ± SEM of 3 independent experiments. ** *p* < 0.01; *** *p* < 0.001 vs. control; ### *p* < 0.001 vs. TGF-β1.

To analyze the contribution of iBETs to mitochondrial ROS production, the mitochondrial levels of superoxide anion (O_2_**.**^−^) were measured using the red fluorescent probe MitoSOXTM in human tubular cells (HK2). The cells stimulated with TGF-β1 and pretreated with iBET JQ1 exhibited lower levels of superoxide anion than the cells only stimulated with TGF-β1 (Figure 3A). In addition, the role of JQ1 in the antioxidant response was analyzed through the gene expression of enzymes such as Catalase, which is involved in the catalytic elimination of hydrogen peroxide, or heme oxygenase-1 (HMOX-1). The mRNA levels of Catalase (CAT) were decreased in the presence of TGF-β1 for 24 h, and pretreatment with JQ1 restored them to basal levels (Figure 3B). In parallel, the expression of HMOX-1 was increased after stimulation with TGF-β1 for 24 h, and JQ1 decreased HMOX-1 to levels even lower than the control (Figure 3C).

### 3.5. JQ1 Favored Mitophagy Induced by TGF-β1 in Human Tubular Cells

**Figure 4:**
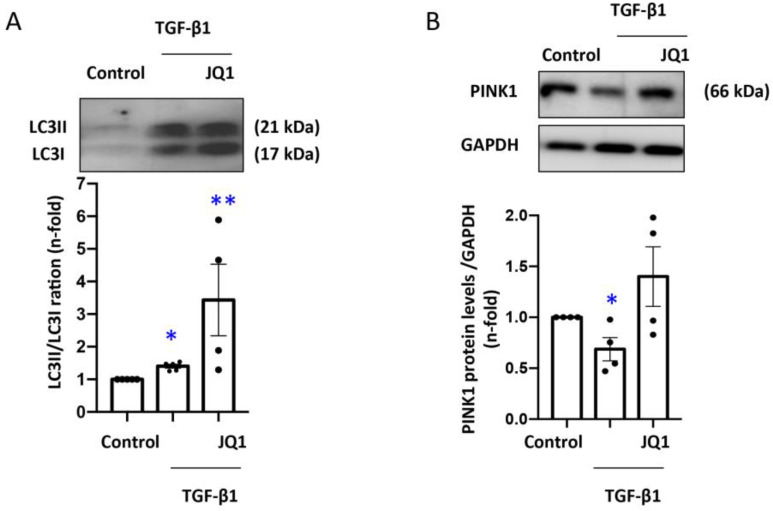
JQ1 maintained the protein expression of mitophagy components in tubular cells stimulated with TGF-β1. Cells were stimulated with TGF-β1 for 24 h and treated with JQ1 (5 μM). Determination of PINK-1 (**A**) and LC3B (**B**) protein levels by Western blot analysis. Data are expressed as the mean ± SEM of 3 independent experiments. * *p* < 0.05; ** *p* < 0.01 vs. control.

To investigate the effects of JQ1 on mitochondrial autophagy induced by TGF-β1 in human tubular cells, we evaluated the protein levels of the key regulators of the mitophagy process, such as PINK1 or LC3B. A 24 h TGF-β1 stimulation in HK2 cells increased the protein levels of the autophagy marker LC3B and reduced PINK1, a mitophagy component characteristic of healthy mitochondria (Figure 4A,B). Treatment with JQ1 favored the mitophagy process by maintaining Map1lc3b/Lc3b levels and preventing the decrease in PINK1 (Figure 4A,B).

### 3.6. JQ1 Prevented the Kidney Downregulation of Electron Transport Chain (ETC) Components in Murine Unilateral Ureter Obstruction

**Figure 5:**
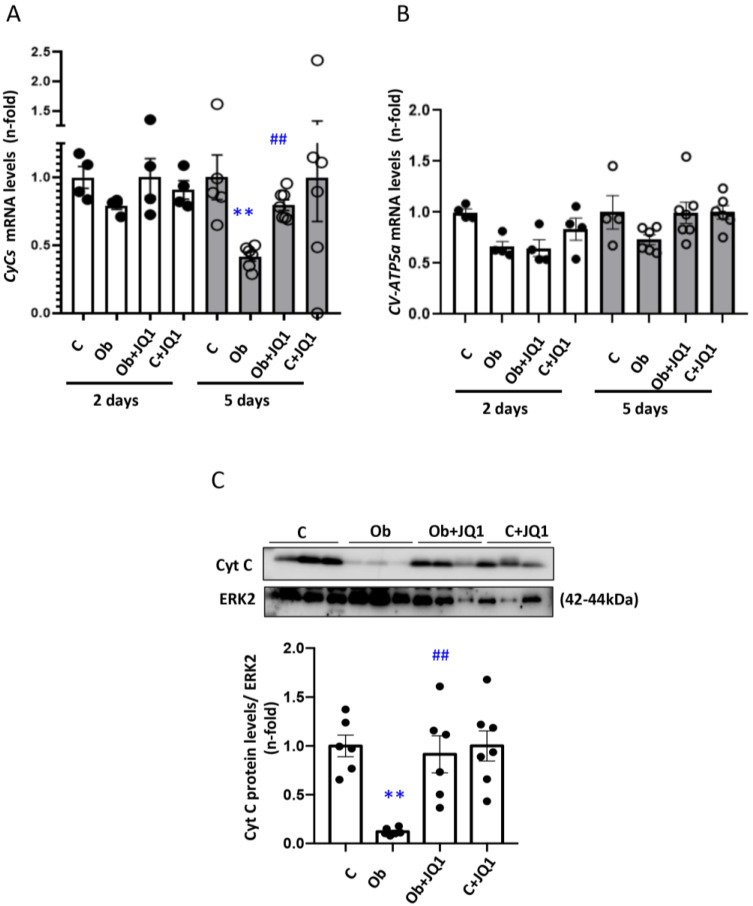
JQ1 prevented the downregulation of components of the electron transport chain (ETC) in Unilateral Ureteral Obstruction (UUO) kidneys. JQ1 prevented the decrease in CytC observed at Day 5 in UUO kidneys. The kidney expression of (**A**) cytochrome C (CytC), (**B**) subunit α of ATP5ase mRNA was determined by RT-PCR. (**C**) CytC protein levels assessed by Western blot analysis. Data are expressed as the mean ± SEM of six to eight animals per group. ** *p* < 0.01; versus contralateral (**C**); ## *p* < 0.01 versus vehicle-treated obstructed (Ob).

After the characterization of JQ1 effects on ETC components in cultured tubular cells, we addressed the in vivo impact in UUO kidneys. In UUO kidneys, Cycs gene expression that codify for Cytochrome C protein decreased at Day 5 kidneys compared to control kidneys (contralateral kidneys) (Figure 5A,B). There was a milder decrease in CV-ATP-5a gene expression (Figure 5A,B). In contrast, no changes in gene expression were observed at Day 2 (Figure 5A,B). JQ1 prevented these changes in gene expression (Figure 5A,B). Kidney protein levels of CytC were also decreased at Day 5 in obstructed kidneys, and this was prevented by JQ1 (Figure 5C).

### 3.7. JQ1 Prevented the Altered Kidney Mitochondrial Dynamics in Murine Unilateral Ureteral Obstruction

**Figure 6:**
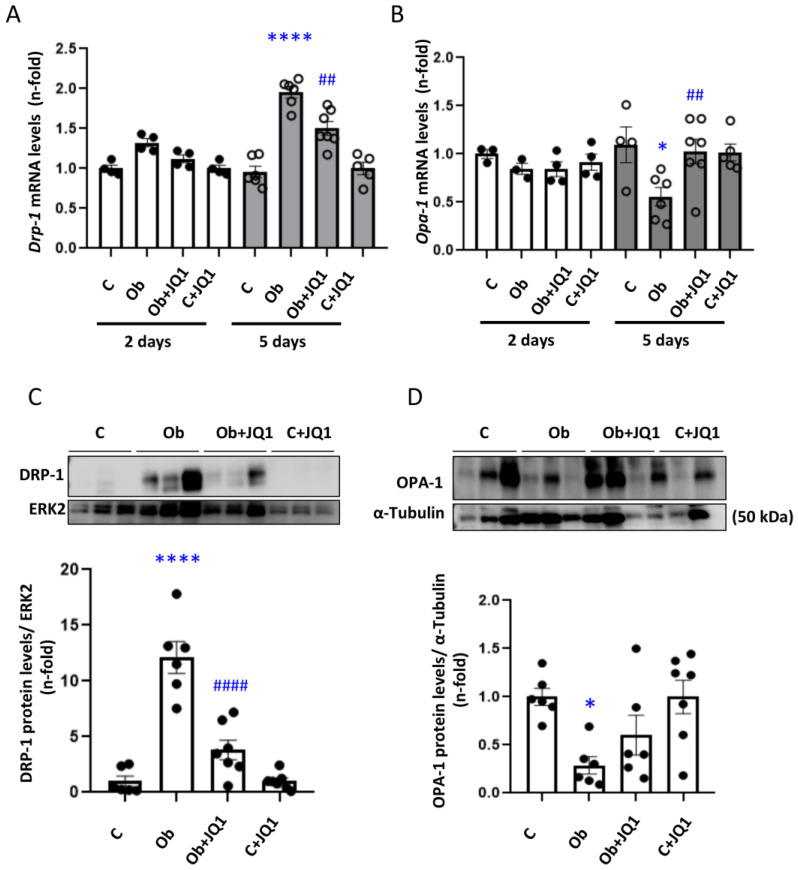
JQ1 prevented the altered kidney mitochondrial dynamics observed in murine Unilateral Ureteral Obstruction. Determination of mRNA levels of regulators of mitochondrial dynamics in UUO kidneys by RT-PCR. The gene expression of (**A**) Dnm1l (dynamin-related protein 1; DRP1) and (**B**) OPA-1 (Optic atrophy type 1) was determined. (**C**) DRP1 protein levels. (**D**) OPA-1 protein levels. Data are expressed as the mean ± SEM of six to eight animals per group. * *p* < 0.05; **** *p* < 0.0001; versus contralateral (**C**); ## *p* < 0.01; #### *p* < 0.0001 versus vehicle-treated obstructed (Ob).

To assess the kidney mitochondrial dynamics in vivo, the gene expression of fission protein DRP-1 and fusion protein OPA-1 was determined. There were no changes in their gene expression two days after ureteral obstruction (Figure 6A,B). However, 5 days after surgery, Dnm1l gene expression levels that codify for DRP1 protein were increased in obstructed kidneys and decreased with JQ1 treatment (Figure 6A). In contrast, the expression of OPA1 decreased in obstructed kidneys, and this was prevented by JQ1 (Figure 6B). The increase in DRP1 and the decrease in OPA-1 protein levels in obstructed kidneys after 5 days was also prevented by JQ1 (Figure 6C,D).

### 3.8. JQ1 Prevented the Kidney Dysregulation of Components of the Antioxidant Response in Murine Unilateral Ureteral Obstruction

**Figure 7:**
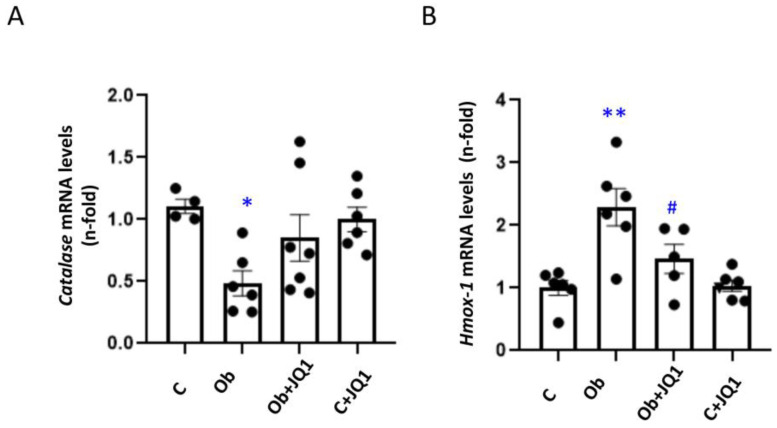
JQ1 prevented the kidney dysregulation of components of the antioxidant response in murine Unilateral Ureteral Obstruction. Determination of mRNA levels of antioxidant enzymes in UUO kidneys by RT-PCR. The expression of (**A**) Catalase (CAT), (**B**) haemoxygenase-1 (Hmox-1) was determined. Data are expressed as the mean ± SEM of six to eight animals per group. * *p* < 0.05; ** *p* < 0.01 versus contralateral (**C**); # *p* < 0.05; versus vehicle-treated obstructed (Ob).

To analyze the antioxidant response in vivo, the expression of enzymes such as Cat and Hmox-1 involved in this response was determined. Kidney catalase gene expression decreased in UUO kidneys after 5 days (Figure 7A), while Hmox-1 gene expression increased (Figure 7B). These changes were prevented by JQ1 (Figure 7A,B).

### 3.9. JQ1 Maintains Kidney Mitophagy in Murine Unilateral Ureteral Obstruction

**Figure 8:**
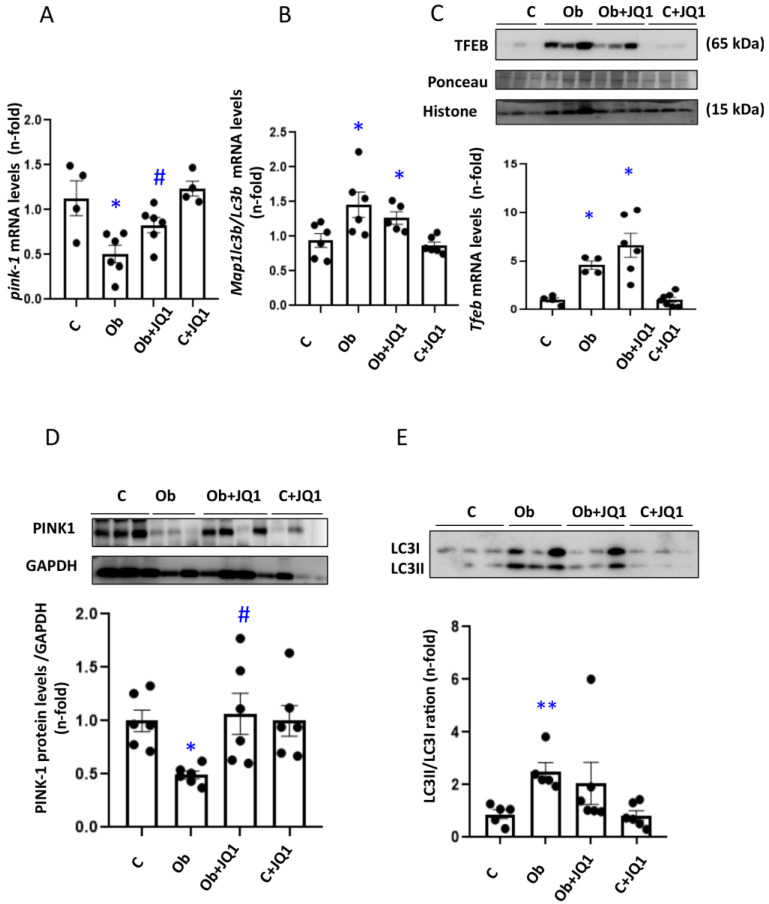
JQ1 maintained the gene and protein expression of mitophagy components in murine Unilateral Ureteral Obstruction. (**A**,**B**) Determination of mRNA levels of mitophagy factors in UUO kidneys by RT-PCR. The gene expression of (**A**) Pink-1, (**B**) Map1lc3b/Lc3b was determined. (**C**–**E**) Determination of TFEB nuclear protein levels (**C**) and total protein levels of PINK-1 (**D**) and LC3II/LC3I ratio (**E**) by Western blot analysis. Data are expressed as the mean ± SEM of six to eight animals per group. * *p* < 0.05; ** *p* < 0.01 versus contralateral (**C**); # *p* < 0.05 versus vehicle-treated obstructed (Ob).

To analyze the contribution of BET inhibition to the mitochondria clearance via the autophagy–lysosome pathway (Mitophagy) in mice, we evaluated the gene expression of the key regulators of the mitophagy process, such as Pink1 or Map1lc3b/Lc3b. The results obtained showed significant decrease in the gene expression (Figure 8A) and protein levels (Figure 8D) of the mitophagy kinase PINK1 5 days after ureteral obstruction, which was partially prevented by JQ1 (Figure 8A,D). Kidney Map1lc3b/Lc3b mRNA levels increased in obstructed kidneys, and this was not modified by JQ1 (Figure 8B). Similar results were observed in LC3B protein levels assessed by Western blot analysis (Figure 8E). In addition, nuclear protein levels of the transcription factor associated to autophagy TFEB were increased in obstructed mice, and this was not modified by JQ1 (Figure 8C).

## 4. Discussion

The kidney is a metabolic active organ that contains high numbers of mitochondria, especially in renal tubular cells [39,40]. Different mitochondrial quality control mechanisms are affected in renal cells during the progression of CKD, and they ultimately lead to the loss of mitochondria functionality [14]. Mitochondria dysfunction is thought to contribute to kidney disease development/progression, and it has been observed in preclinical models of CKD [9,15]. Indeed, mitochondrial damage markers, such as urinary mitochondrial DNA (mtDNA), could be used as an kidney injury biomarkers [41]. Currently, pharmacological drugs targeting mitochondrial damage has been investigated to ameliorate renal damage [42]. The data presented here shows that the iBET JQ1 improve mitochondrial dynamics, functionality, and oxidative stress, as well as mitophagy induction in kidney disease, supporting the importance of the maintenance of mitochondrial integrity in progressive kidney diseases.

In fact, several preclinical studies have shown that BDR4 regulates pro-inflammatory and pro-fibrotic genes [25].The iBETs JQ1 that specifically inhibits BRD4 has been previously demonstrated to inhibit proinflammatory gene transcription in renal cells and in experimental kidney damage [28]. The data presented here show that JQ1 regulates many mitochondrial-related genes. These action mechanisms could be related to the beneficial effects of JQ1 in the maintenance of mitochondrial integrity.

BET protein inhibitors are being studied as modulators of mitochondrial injury in renal and cardiovascular diseases [32,43]. A seminal study suggests that BET proteins could modulate mitochondrial genes. In a high-throughput chemical screen in human cells with mitochondrial Complex I Mutations, the use of iBET I-BET525762 correlated with an increase in OXPHOS, associated to increased mitochondrial chain complex I expression [44]. A transcriptomic analysis of heart tissue from a BRD4 knockout mouse identified an early and specific disruption of essential genes to mitochondrial energy production and homeostasis such as ETC components [33]. OXPHOS is reduced in CKD; the cause is not well understood [45].

The ETC is composed by different protein complexes that participate in OXPHOS [46]. The flow of electrons in ETC complexes can generate reactive oxygen species (ROS). Excessive ROS production by complexes I and III may trigger an uncoupling of ETC components causing a decrease in ATP production and the malfunctioning of mitochondrial respiration [2,47]. Cytochrome C is key ETC component whose release triggers apoptosis [15]. Decreased expression of cytochrome C is associated to a reduced OXPHOS rate [48]. In our in vivo study, kidney obstruction for 5 days resulted in a significant decrease in cytochrome C gene and protein levels in comparison to control mice. Furthermore, gene expression levels of complexes CII-SDHB and CV-ATP-5a were decreased in 5 day-obstructed kidneys. In addition, in cultured tubular cells, TGF-β1 also decreased the protein levels of the CV-ATP-5a complex after 24 h. Pretreatment with JQ1 prevented the in vivo and cell culture changes.

Mitochondrial dynamics, i.e., the balance between fusion and fission, contribute to mitochondrial health. Unbalanced mitochondrial dynamics contribute to the progression of CKD [17]. In our study, we observed an increase in the expression of the fission GTPase DRP-1 that drives mitochondrial fragmentation. In various CKD models such as subtotal nephrectomy [49] or diabetic nephropathy [50], DRP-1 is overexpressed. In prostate cancer, BRD4 inhibitors block mitochondrial fission [51]. In our study, JQ1 prevented the changes in kidney DRP-1 gene and protein expression observed 5 days after unilateral ureteral obstruction. Moreover, TGF-β1 increased DRP-1 protein levels in cultured tubular cells, and this was also prevented by JQ1. By contrast, the expression of OPA-1, a protein involved in mitochondrial fusion [17], was decreased in different CKD models including subtotal nephrectomy [49] or the adenine model [52]. We also observed a decrease in OPA-1 gene and protein expression in obstructed kidneys after 5 days, which was also prevented by JQ1. Similar restoring effect of JQ1 treatment in OPA-1 levels was observed in human tubuloepithelial cells (HK2) stimulated with TGF-β1.

Mitochondrial injury decreases mitochondrial viability [53]. The number of kidney cell mitochondria is decreased by insults such as high glucose concentrations or iodinated contrasts [54,55], as well as by TGF-β1 [56]. Our results using the MitoTracker^TM^ probe show that stimulation with TGF-β1 decreases mitochondrial viability; this was prevented by JQ1. One of the mechanisms related to the loss of mitochondrial functionality is the loss of its membrane potential (∆Ψm) [7]. In CKD patients, an uncoupling in the respiratory chain had been observed establishing a relationship between renal damage and decreased ∆Ψm [57]. In our in vitro experiments, the staining of the human tubular cells with TMRM (tetramethyl–rhodamine) disclosed a loss of membrane potential in cells stimulated with TGF-β1 that was prevented by JQ1.

The ROS are produced by mitochondria during the normal functioning of the respiratory chain and OXPHOS, resulting in ATP production. Excessive ROS production or deficient scavenging in pro-oxidant, anti-oxidant, or redox-sensitive signaling pathways results in oxidative stress and cell damage [58]. Reactive oxygen species (ROS) are fundamental mediators for numerous cellular processes in homeostasis such as growth, survival, or proliferation [59]. However, oxidative stress may cause mitochondrial dysfunction and kidney disease [59,60,61]. Conceptually, dysfunctional mitochondria cause mitochondrial oxidative stress (mtOS); however, oxidative stress may also stress the mitochondria [62]. BRD4 regulates signaling pathways that control oxidative response [63,64]. In this context, we hypothesized that JQ1 also regulate the transcription of several genes associated to antioxidant response.

Previous studies suggested that JQ1 treatment prevents H_2_O_2_-induced intracellular reactive oxygen species production, and the chromatin–immunoprecipitation analysis showed the recruitment of Brd2 and Brd4 to nuclear factor erythroid 2-related factor 2 (Nrf2)-binding sites on the promoters of heme oxygenase-1 and NADPH quinone oxidoreductase 1 [64]. Moreover, the inhibition of BRD4 in chondrocytes ameliorates oxidative stress-mediated apoptosis and cartilage matrix degeneration by the modulation of the Nrf2-HO1 signaling pathway [65]. Our in vitro and in vivo studies showed an increase in oxidative stress from mitochondrial origin, including superoxide assessed by the mitoSOX^TM^ assay in tubular cells exposed to TGF-β1. JQ1 pretreatment restores mitoSOX^TM^ fluorescence signal to control values. In addition, we observed a decrease in gene expression levels of Catalase (CAT), which was prevented by JQ1 in mice and cells. CAT mediates hydrogen peroxide (H_2_O_2_) elimination and protects the kidneys from oxidative stress [66].

In addition, studies in mice have described the role of JQ1 in ROS production. A study in cisplatin nephrotoxicity described that JQ1 enhanced the protein expression of antioxidant factors including NRF2 and heme oxygenase-1 (HMOX1) while diminishing the expression of the nitrosative protein inducible nitric oxide synthase (iNOS) [67]. More focused in obstructive nephropathy, a study in rats described that Brd4 regulation with jQ1 diminished Nox4-induced ROS. The authors observed that JQ1 significantly suppressed UUO-induced hydrogen peroxide production. In this manuscript, it was also demonstrated that JQ1 diminished hydrogen peroxide production in HK2 cells stimulated with TGF-β [68]. No other study of kidneys so far has focused on the analysis of BET inhibitors in the oxidative response. Previous data showed that HMOX-1 expression increases in response to cadmium and is a marker of oxidative stress [69]. In CKD, increased HMOX-1 prevents kidney damage [70]. In our study, obstructed kidneys and renal cells showed increased HMOX-1 levels, and this was prevented by JQ1.

All these results and previous studies corroborated the possibility that JQ1 directly modulates the transcription of these antioxidant response genes, contributing to mitochondrial oxidative stress and damage.

Mitophagy degrades damaged mitochondria via autophagosomes and the subsequent transference of the content to the lysosomes [71,72,73]. Defective mitophagy has been related to human disease, including aging, cancer, cardiovascular disease, as well as renal disease [16,74,75,76]. The PINK1-PARK2 pathway regulates mitophagy [14,77,78]. In diabetic cardiomyopathy, JQ1 improved mitochondrial function and restored cardiac function and structure via PINK1/Parkin-mediated mitophagy activation [32]. Our results in the UUO model showed an increased gene and protein levels of mitophagy factors such as LC3 and TFEB, the transcription factor that modulates autophagy. In addition, kidney PINK1 levels were reduced 5 days after obstruction. JQ1 increased PINK1 levels that induce PARKIN1 recruitment and autophagosome formation, producing a milder impact on LC3 and TFEB levels. Similar results were observed in human tubular epithelial cells exposed to TGF–β1 and treated with JQ1. All these signaling modulations favor the degradation of damaged mitochondria and restoration of mitochondrial damage.

## 5. Conclusions

**Figure 9:**
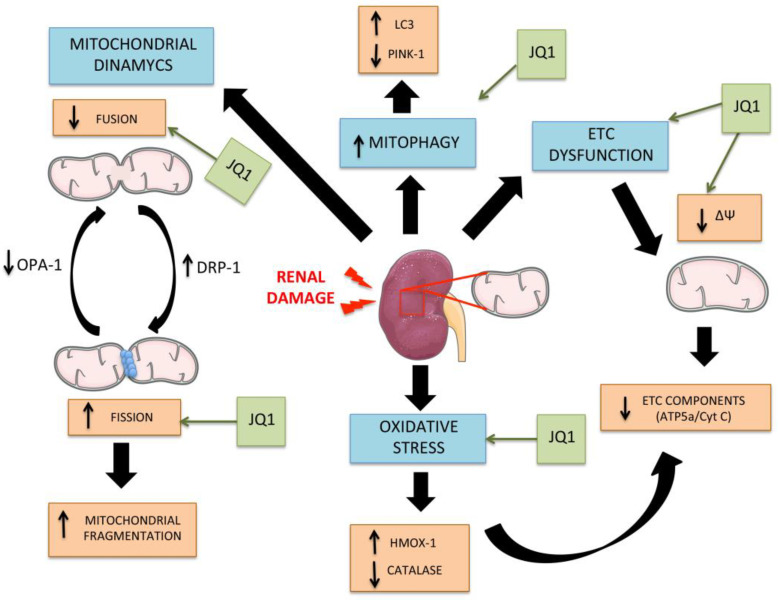
Mechanisms involved in mitochondrial damage during kidney damage and JQ1 beneficial effects. As a consequence of the initial damage, the mitochondria lose quality control mechanisms, triggering oxidative stress due to excess ROS production, deregulation of mitochondrial dynamics favoring fusion and consequent fragmentation, dysfunction of the electronic transport chain and loss of membrane potential levels (∆Ψ), and other mechanisms, such as mitophagy. JQ1 exerts its beneficial effects restoring the loss of electron transport chain components (ATP5a/Cyt C), modulating mitochondrial dynamics components (DRP-1 and OPA-1) and antioxidant response genes (CAT and HMOX-1), and maintained the mitophagy response to damage (LC3/PINK1).

During CKD progression, multiple inter-related disruptions in mitochondrial function and control can be observed. An excess of ROS production is related to unbalanced mitochondrial dynamics, mitochondrial membrane potential changes and mitochondrial degradation. The end result is mitochondrial injury and loss of mitochondrial functionality, therefore contributing to kidney disease. Epigenetic drugs such as iBET, and specifically JQ1 as we shown here, favorably modulate multiple aspects of mitochondrial function and control, including a better control of oxidative stress as well as regulation of mitochondrial dynamics, functionality and degradation (Figure 9).

## Data Availability

Not applicable.
